# ProvOER model: A provenance model for Open Educational Resources

**DOI:** 10.1016/j.heliyon.2023.e13311

**Published:** 2023-02-02

**Authors:** Renata Ribeiro dos Santos, Marilde Terezinha Prado Santos, Ricardo Rodrigues Ciferri

**Affiliations:** Federal University of São Carlos (UFSCar), São Carlos, Brazil

**Keywords:** Open Educational Resources, Data provenance, Provenance model, Metadata

## Abstract

Open Educational Resources (OER) can be adapted and combined to create new resources that better meet the specific needs of different kinds of users and scenarios. In this sense, OER strongly contributes to generating and sharing educational knowledge. Due to the possibility of creating a new OER through the revision and remix activities, the original OER and the transformation process should be adequately identified. This way, the user of the OER has enough information about the history of the resource and, thus, can use it with confidence and security. In this context, determining data provenance, which describes the history of a data from its origin to its current state, becomes very relevant. For OER, there are examples of metadata standards and digital repositories that help to obtain the data provenance. However, the information collected is insufficient to identify the entire history of the provenance of OER. This article proposes a Provenance Model for OER called the ProvOER Model, which allows the documentation and identification of the provenance of OER. For this purpose, a minimum set of metadata was defined that reflects the OER intrinsic properties and the activities that created a new OER. The experiments showed that the ProvOER Model produced a suitable representation of the provenance of OER. In addition, the ProvOER Model allowed identifying the original OER used in a revise or remix activity and the continuous stretch used to create a new resource.

## 1 Introduction

Open Educational Resources (OER) are teaching, learning, and research resources that reside in the public domain or have been released under an open license. These resources encompass any material, software, or tool that can be used to support access to knowledge [1]. OER are commonly available as text, textbooks, articles, thesis, slides, animations, multimedia applications, audio, classes, complete courses, exercises, images, games, course modules, podcasts, quizzes, simulations, and videos [2], [3], [4]. In this article, we focus specifically on the text scope.

Due to the characteristics of legal openness, OER can be **r**eused unchanged in different contexts, **r**evised (adapted) and/or **r**emixed (combined) to create new resources, **r**edistributed to third parties and **r**etained as a copy [5]. The possibility of carrying out these activities expands the opportunities for the educational use of OER, mainly because these resources can effectively be revised and/or remixed to create new educational resources.

Creating new OER from different OER opens space for several questions about the created new resource. For example: *What is the original OER?*, *Who is responsible for the original OER?*, *How was the process used to create the OER?*, *What was the revision carried out in the creation of the current OER?* and *Which OER were combined to create the new OER?* In this sense, the problem identified is that the OER lack information about the different transformation processes that resulted in the current resource. Thus, the possibility of documenting this information enhances the reuse, revision, and/or remix of OER since the user can access enough information to use the OER with safety and confidence.

In this sense, the data provenance, which describes the history of a data from its origin to its current state, becomes important for documenting how an OER was created. The data provenance is usually stored as a set of metadata. Therefore, the metadata must be adequate and sufficient and reflect the particularities of the context in which the provenance information will be described. For an OER created through revision, remix, or both activities, it is essential to consider how the characteristics inherent to each activity influence the documentation of the OER provenance.

As a justification, it is important to highlight that in the literature, no examples of related works focus their efforts on studying the provenance of OER. On the other hand, in the context of educational resources, there are examples of metadata standards that consider provenance information as descriptive data. However, metadata defined for this purpose are optional and may not be completed, which is a barrier to documenting provenance information. In addition, they do not consider the particularities related to creating an OER through revision, remix, or both. We can also point out examples of digital repositories that stimulate and record the provenance of OER. However, they present limitations regarding the complete documentation of the transformation processes that occurred in the history of an OER.

Due to the lack of works related to the theme of the provenance of OER and the weaknesses presented by metadata standards and digital repositories, we observed the possibility of contribution since there is no minimum set of provenance metadata for the documentation of the history of OER. Thus, the research question that guides this article is *What is the minimum set of metadata that must be documented to describe the provenance of OER?* The purpose of this article is to present the Provenance Model for Open Educational Resources (ProvOER Model) for the documentation and identification of the provenance of OER. The ProvOER Model is structured according to the Entity-Relationship Diagram, which makes it possible to represent all the components and metadata relevant to the description of the OER history. In this sense, we defined a minimal set of metadata that reflects the particularities of OER and the different transformation processes that can be carried out to create a resource. Thus, through the ProvOER Model, it is possible to identify and monitor the successive activities carried out to create a new OER.

The study presented in this article is theoretical since it aims to explain and present a minimum set of provenance metadata for OER. In this sense, validating the ProvOER Model focuses on a case study in which it is possible to present the provenance metadata and the transformation processes that resulted in a new OER. However, we emphasize that the ProvOER Model can be used semi-automatically in other scenarios, such as a digital repository, as explained in Section 6.

This article is organized as follows: 2 presents the methodology used to propose the ProvOER Model. 3 describes the theoretical foundation related to OER and data provenance. 4 presents examples of metadata standards and digital repositories that allow the description of OER provenance. 5 details the proposed ProvOER Model. 6 presents the results and discussion of validating the ProvOER Model. Finally, 7 presents the conclusion and future work.

## 2 Materials and methods

This section presents the methodology used to create the ProvOER Model. We studied the theoretical foundation of OER and data provenance to understand the particularities of these concepts. We identified a gap in the literature regarding efforts to describe the provenance of OER since there are no contributions related to this issue. We also identified metadata standards and digital repositories that allow the description of the provenance of OER, but they have some weaknesses. Thus, as a contribution, we developed the ProvOER Model for documenting and identifying the provenance of the OER.

The ProvOER Model reflects the intrinsic characteristics related to the OER and the transformation process used to create a new resource. And, therefore, can be used to document the history of an OER. To validate the ProvOER Model, we carried out a case study by presenting an example of an OER created through review and remix. We demonstrated how the particularities of these activities are documented in our model and how the provenance of the OER created can be analyzed. We used the Oracle Live SQL^1^ relational database to create the tables and store the metadata registered by the ProvOER Model.

## 3 Open Educational Resources and data provenance

OER are an important feature for creating and sharing educational knowledge. Openly distributing educational resources increases their reach and impact by allowing other users to adapt the resources to their specific needs [6].

These educational benefits are possible by open licenses, in which the copyright holder of the OER grants third parties permission to access, reuse, repurpose, adapt and redistribute the resources [7]. Compared to copyright, using a more permissible and flexible license expands the possibilities of the legal use of OER. In this sense, five activities (“5Rs”) can be carried out with the OER [5]. These activities are:

• Reuse – use of an original OER;
• Revise – adaptation of an OER, such as insert of content, remove or translation;
• Remix – combination of two or more OER;
• Redistribution – sharing an OER;
• Retention – creation and control of copies of an OER.

The main example of open licenses for OER is those established by Creative Commons (CC).^2^ Through these licenses, the OER's copyright holder may grant permission to third parties to adapt, combine, and/or commercially use resources. CC licenses are recognized as an international standard but not exclusive. As other examples, it is possible to cite the GNU General Public License (GNU GLP),^3^ suitable for the software, and the Open Data Commons (ODbL),^4^ convenient for database licensing. It is essential to consider that digital repositories and other organizations that provide OER have the autonomy to define and use an exclusive open license.

In addition to the characteristic of legal openness, other aspects can be highlighted for OER. To facilitate the use of digital OER, it is advisable to make them available in open file formats, which can be easily manipulated by any software [3]. Using an open file format increases the possibility of revising and remixing OER [8]. Furthermore, OER can be in any media, digital or not, and format [3], [9], [7]. Moreover, they are made available at no cost to users [2], [9], [4], [7]. Another consideration is that an OER can be composed of different educational resources [1], as, for example, texts and images can be used to create a textbook. In this sense, the resources used to create an OER may have a specific open license [10].

New OER can be created “from scratch” or by revising and/or remixing an original OER. In a life cycle, OER are usually stored in digital repositories, which are spaces that facilitate the sharing and retrieval of educational resources [11]. OER stored in digital repositories are usually described by metadata, which can be used to search, evaluate, acquire and use a resource [12]. The metadata can be arranged in a standard. One metadata that can be collected for the description of an OER is data provenance.

The word provenance means origin or source [13]. Data provenance is a set of metadata that describes information about the history of data, from its origin to its current state. Furthermore, it encompasses the storage of the entire process used to produce the data, such as data transformation and the data workflow.

There are several motivations for describing the data provenance [14], for example, evaluating the quality of data based on its origin and the transformation processes carried out to generate the data. Another application is auditing since it is possible to trace the history of data and identify potential errors caused during a transformation process. In addition, it can be used to determine the person responsible for the data, which allows the attribution of responsibility and alerts about possible errors. The detailed description of the data provenance information also enables the reproduction of experiments since the provenance information describes all the steps performed to generate new data. In addition, it can be used to understand the data.

For data provenance, it is important to consider what provenance metadata must be collected, how it should be collected, where it can be stored, and how this information should be presented to the user. In the first case, no single and fixed answer determines the most relevant metadata for the description of the history of data. This information is conditioned to the context for which it is analyzed. For a relational database, for example, the original data set that contributed to the creation of new data (why-provenance) and the location in the database of the data that effectively contributed to this purpose must be identified (where-provenance) [15]. In geographic data, the capture scale, acquisition date, and coordinate reference system are metadata that must compose the provenance information [16].

After identifying the provenance metadata to be stored, it is important to establish a strategy to collect this information manually, semi-automatically, or automatically. That is, with or without user intervention. The manual collection is time-consuming, error-prone, and sometimes incomplete [17]. Subsequently, this information can be stored with the data or separately. Finally, the data provenance can be made available, for example, through descriptive metadata.

## 4 Metadata standards, digital repositories and provenance

In this section, we present examples of metadata standards and digital repositories that make it possible to describe the provenance of OER. We must point out that we did not identify contributions in the literature related to provenance in the context of OER.

Commonly highlighted metadata standards for describing educational resources are the Dublin Core Metadata Element Set (DCMES) [18] and the IEEE Learning Object Metadata (IEEE LOM) [19]. The DCMES standard (version 1.1) or “Dublin Core” was developed to describe any digital resource. This standard includes fifteen optional elements *(contributor, coverage, creator, date, description, format, identifier, language, publisher, relation, rights, source, subject, title, and type)* that can be used to detail the description of OER. In addition, they are generic, which makes it possible to describe a wide variety of resources, such as OER, but limits the documentation of relevant aspects in particular contexts. A set of terms is also established to complement the fifteen elements and describe different digital resources.

One of the elements considered in Dublin Core explicitly related to the data provenance is *source*, which corresponds to the identification of the resource from which the described resource was derived. In the case of OER, this information corresponds to the original OER used in a revision and/or remix activity. Another relevant element is *relation*, which identifies the relationship between a resource and the target resource. This type of element can take on different values, such as “is part of”, “is a version of”, and “has part of”. The term *provenance* should also be noted. This term corresponds to “a statement of any changes in ownership and custody of the resource since its creation that are significant for its authenticity, integrity, and interpretation” [18]. Although it is named provenance, the term relates to the author and copyright holder of the resource and not effectively the history of an OER.

The IEEE LOM metadata standard (version 1.0) is based on Dublin Core and was developed for the description of Learning Objects (LO), which are digital resources that can be reused to support learning [20]. This standard is organized into nine categories *(General, Life Cycle, Meta-metadata, Technical, Educational, Rights, Relation, Annotation, and Classification)*, formed by optional metadata. As they are directed to LO, the metadata considered in the IEEE LOM are specific to describe resources used in the educational context.

The IEEE LOM describes the data provenance in the category *Life Cycle*, which stores information about an educational resource's history and current state. The metadata specified for this category is version, status (draft, final, revised, or unavailable), and contribute. The latter corresponds to the natural or legal person who contributed to the resource, such as the author, publisher, and editor. We can cite the category *Relation* as a complement to the life cycle description.

In Dublin Core and IEEE LOM, the possibility of describing the relation between two OER is essential to understanding how an OER was formed from an original OER. However, it is impossible to specify how the original OER was used and which elements make up the current OER. This limitation is a barrier to documenting a remix activity. For example, pointing out that an OER B “is part of” an OER A is not enough for the user to identify which parts of OER A were used to create OER B. Furthermore, as these standards are not specific to OER, it is not maintained information to identify a revision in the original OER. Another weakness is that the metadata established in these standards are optional and, therefore, may not be filled in, which makes it impossible to identify the provenance.

We can also cite the Learning Resource Metadata Initiative (LRMI),^5^ which specifies a set of classes and properties for describing educational resources. One of the properties that make up the LRMI is *Is Based On*, which corresponds to “a resource from which this work is derived or from which it is a modification or adaptation”. This information is relevant for identifying the original OER but insufficient for the complete documentation of the provenance of OER.

Digital repositories whose metadata are organized according to the Dublin Core or IEEE LOM standard also allow provenance documentation. In addition, there are examples of digital repositories that are not organized according to these standards but also make it possible to describe the history of OER. One example is Connexions, currently maintained by OpenStax.^6^ Connexions was an OER repository in which resources were licensed through a CC license. OER were described by metadata, such as author, copyright holder, and license. Although disabled, this repository was an important example of a digital environment in which new OER could be created from the resources stored in the repository. In addition, this action was documented, and the information was available to OER users.

The two strategies adopted in Connexions to describe the provenance of OER were version history and the possibility of creating derivative works. A version number was automatically generated for all OER stored in the repository. When the resource was changed, the previous version of the OER was overwritten by the current one, and the version number was incremented. All changes made to an OER were documented, including those responsible for carrying out this activity and the date on which this occurred. The description of the change was succinct but pointed out the difference between versions. Also, the OER available to the user matched the current version of the resource, but previous versions could be consulted via links. The ability to access earlier versions of a resource is important so that the user understands the difference between resources. Another advantage is the possibility of accessing a version more suitable for their context.

The derivative work was another strategy in which a copy of the original OER was adapted. So this resource was not overwritten. The adaptation was indicated in a generic way, such as “translation” or “alteration of the author's material”. In addition, the original OER was explicitly identified, in the current OER, through the title, author, and URL. A weakness that can be pointed out is that it is impossible to document the remix activity among two or more original OER since only one resource can be derived. Another limitation shared for the version history is the impossibility of identifying the localization in the original OER where the changes were made.

Another example of a digital repository is the OER Commons.^7^ In this repository are stored OER and educational resources subject to copyright restrictions. Most OER are licensed through a CC license. In OER Commons, OER are described by metadata, such as title, language, and condition of use, which correspond to the type of CC license assigned to the resource.

Depending on the type of open license assigned to a resource, the revision of an OER stored in the repository is allowed. For the created OER, a version history is maintained, in which it is possible to view the title of the original OER, the author, and the creation date of this resource. In addition, for the original OER are registered, the title, author, and creation date of the OER created from it. The possibility of documenting the original OER is essential for identifying the data provenance. However, in OER Commons, it is impossible to determine what changes were made to create a new resource. Also, the remix activity cannot be adequately documented, as only the transformation of an OER can be recorded, and the remix activity involves at least two resources.

We noticed some weaknesses common to Connexions and OER Commons digital repositories. The inability to identify and locate the changes made in an original OER and the impossibility of documenting the remix activity among two or more resources are barriers to the complete description of the provenance of OER.

## 5 ProvOER model

**Figure 1:**
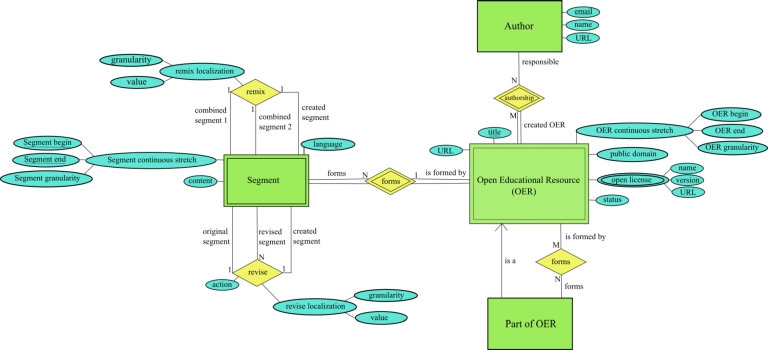
Provenance Model for Open Educational Resources (ProvOER Model).

In this section, we present the ProvOER Model, a Provenance Model for Open Educational Resources that allows the documentation and identification of the provenance of OER. Fig. 1 presents the conceptual scheme of the ProvOER Model. We use Entity Relationship diagram notation, which is close to the notation of the original proposal of [21] according to [22].

The core element of the ProvOER Model is the Open Educational Resource (OER). This type of weak entity is instantiated when a new resource is created “from scratch” or through a revision and/or remix activity. For an OER, it is essential to maintain its title to identify the resource. As a good practice, we consider that the title should be concise and objectively reflect the purpose of the OER. In the ProvOER Model, we also maintain the size of the continuous stretch, with the beginning and the end, which delimits the full OER. This article focuses on textual OER, so the granularity defined for the continuous stretch that delimits an OER is page.

In addition to this metadata, we identify whether an OER is in the public domain or licensed through an open license. In the case of the public domain, we consider this option as a flag, which can assume the values *yes* or *no*. For an open license, we store the name, version, and URL for more information about the license. These data make it possible to identify the license to use, as each type of open license has specific permissions and limitations. Furthermore, we need to consider that an OER may be subject to more than one open license. In this case, it is up to the user to choose which type of open license will be adopted [23]. For example, a video game soundtrack can be licensed under a license CC and GNU GPL [23].

In the ProvOER Model, an OER is linked to the author, who is the person responsible for authoring one or more resources. This action can be performed individually or collaboratively. For the author, we recommend storing information that allows identification and contact. So we keep the email, name, and URL as attributes. An author is usually associated with an individual, such as a teacher, instructional designer, or student, but can also be a legal person, such as an organization, university, or research institute. In the ProvOER Model, we do not distinguish between a natural and legal person since the data collected for a person are general and are present in both scenarios.

For purposes of provenance, the information about the author is one of the aspects that can be considered for identifying the source of the OER. The other elements are the URL for the resource's digital location and the original OER. For an OER created “from scratch,” the author identification and URL are the only information available to determine where the OER came from. In the case of an OER created through adaptation (revision activity) and combination (remix activity), we can also consider the original OER used as the basis for creating a new resource. In this sense, the author and URL of the original OER must also be stored. Thus, we keep the resource URL. However, in the ProvOER Model, we recommend storing the OER and its contents together so that any transformation process can be adequately documented.

An OER can be made up of other educational resources with different open licenses. We consider the elements Part of OER and Segment to represent this particularity. Both correspond to a continuous stretch inside an OER, with a beginning and an end. However, a Part of OER is understood as an OER as it has a specific open license. Thus it is independent of the full OER. A Segment does not have its own existence, as it is necessarily linked to the OER that contains it and, therefore, is subject to the same open license as the full OER.

In the ProvOER Model, we document where the revise and remix activity took place in the original OER. Thus, the granularity of an activity can be page or integral OER when a resource is used in its entirety. For example, an original OER can be submitted to review activity on page 11. This information is important to locate precisely where the transformation process occurred.

An OER can be composed of one or more Parts of OER. Since a Part of OER is an OER, all metadata maintained for an OER are also collected for a Part of OER. Also, due to this consideration, other Parts of OER can form a Part of OER. In addition, the open license assigned to the full OER does not coincide with the license of each Part of OER contained in the resource. One situation that should be considered is revising and/or remixing copyrighted educational material. In this case, for the Creative Commons guidelines [10], the user must ask the OER copyright holder to license the resource under an open license. In this case, if the open license assigned to the educational resource differs from the full OER, the resource is stored as a Part of OER.

As a restriction of the ProvOER Model and for provenance, we established that an OER is formed by at least one Segment, which corresponds to the complete OER. A Segment is instantiated when an OER or Part of OER is created. Another situation is when a specific Segment is revised and/or remixed. In this sense, we restrict and associate these activities to a Segment so that it is possible to track the provenance information. It is essential to note that a Part of OER is also formed by at least one Segment. In the ProvOER Model, a Segment is associated with a single OER, which makes it possible to identify the origin and authorship of the Segment.

For a Segment, we keep the continuous stretch that delimits the beginning and end of the Segment in an OER. For provenance purposes, this information is essential for identifying and locating the adapted Segment in a revise or combined in a remix. We also keep the granularity referring to that stretch. For the scope of this article, the granularity of a Segment can be a line, page, or chapter. In the ProvOER Model, we also maintain the Segment content. As pointed out, we recommend keeping the OER content and metadata together for provenance control purposes.

Other aspects about Part of OER and Segment can be pointed out when we consider the inherent particularities of creating an OER “from scratch” or through revise and/or remix activities, as explained below:

### 5.1 OER created “from scratch”

An OER created “from scratch” features original content created solely through the intellectual contribution of one or more authors. That is, it does not come from another OER. In the ProvOER Model, we consider that the new OER can be composed of Segments, Parts of OER, or Segments and Parts of OER.

In the first case, the OER is composed of one or more Segments. In this sense, according to the open license assigned to the OER, any Segment can be used by third parties to create a new OER. In other words, it is not the role of the OER author to define which Segments make up the OER but third parties, who are free to choose the most appropriate continuous stretch to compose a resource. A third party corresponds to one or more authors responsible for creating a new OER by adapting and combining an original OER.

In the second case, the OER comprises two or more Parts of OER. This way, we consider that the OER is generated “from the outside in”. First, the integral OER is created; later, this resource is divided into Parts of OER with specific open licenses. Thus, the OER author is also responsible for creating the Parts of OER. This scenario allows the copyright holder to define how third parties should use the Parts of OER. For example, a Part of OER may be submitted to a restrictive open license that does not allow adaptations. We consider that the copyright holder must be free to decide how the OER can be used and have the autonomy to choose an open license that reflects his needs. We emphasize that the ProvOER Model does not adopt or recommend any specific open license.

In the third case, the OER is formed by one or more Segments and one or more Parts of OER. This way, the copyright holder can license one or more Parts of OER with a specific open license. In contrast, the remaining Segments share the same permissible license as the full OER.

### 5.2 OER created through a revise activity

In the ProvOER Model, the revision activity consists of adapting one or more Segments. That is, an original Segment is revised, which results in a new Segment. The original and created Segments correspond to the original OER and the created OER. It should be noted that the original Segment and the created Segment are different since, for provenance purposes, the original OER is not overwritten. We emphasize that the possibility of revising an OER is conditioned to the permissions associated with the open license.

We consider that the revision of an OER results in a new resource. This consideration also extends to a Part of OER. Thus, revising a Part of OER creates a new Part of OER. In this sense, the created Part of OER may be licensed under the same open license assigned to the original Part of OER or under a different permissible license. In the latter case, the open license of the integral OER may coincide with that of the Part of OER. Thus, as an exception, a Part of OER created through revise may be licensed through the same open license as the OER that contains it.

Different revise actions can change one or more Segments from an OER. So, to allow the description of the OER history, we keep the name of the revise action performed for the change of a Segment. Based on the elements of the ProvOER Model, we present the possible revise actions for a text:

• **Insert**

This revise action consists of inserting one or more Segments. In this case, the inserted Segment is new and was created by the author of the new OER. In the ProvOER Model, the insertion of a Segment from another OER is understood as a remix.

**Figure 2:**
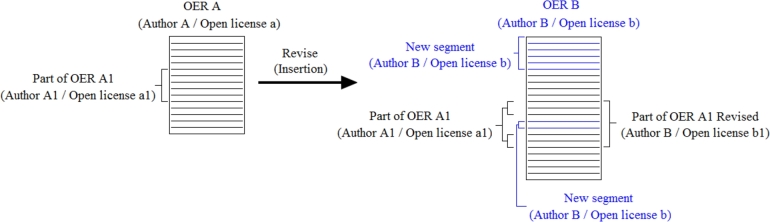
Example of insert action for creating a new OER. OER B was created by inserting the Part of OER A1 from the original OER A. Author B is responsible for creating the OER B and the continuous stretch in blue, which correspond to new content.

Fig. 2 shows an example of insertion. Original OER A was created by author A and is licensed under an open license called a, which allows third parties to change the resource. The OER A is formed by one or more Segments and a Part of OER named A1. This Part of OER was created by author A1 and is licensed through an open license named a1. The author of OER A and Part of OER A1 may be different, as OER A may have been created “from scratch” along with Part of OER A1. Another possibility is the creation of the OER through revision, remix, or both. In the latter case, the Part of OER A1 would come from another resource.

OER B was created from the insertion of new Segments in OER A. In this sense, OER B and the new Segments were created by the same author and are licensed through the same open license. The insertion action can be performed in any position of an OER. In the example, a new Segment was inserted at the beginning of OER B and in the middle of Part of OER A1, which resulted in Part of OER A1 Revised. Authorship of the new Part of OER is also attributed to author B, while Part of OER A1 retains the original Segments created by author A1. The author of Part of OER A1 Revised is free to assign the most appropriate open license for this new resource.

• **Remove**

This revise action consists of removing one or more Segments.

• **Replace**

Replacement is equivalent to exchanging one or more Segments from an original Segment. We reinforce that, in the ProvOER Model, replacing a part of OER is analyzed as a remix. This revised action can also be understood as updating content.

• **Translate**

Translation consists of changing the language of one or more Segments. Other revised actions can follow this action, for example, the removal or replacement of cultural terms that do not apply to a given context. In the ProvOER Model, we keep the Segment language.

### 5.3 OER created through a remix activity

In the ProvOER Model, the remix activity consists of the combination of two or more Segments. Two or more original Segments from different OER are combined to create a new Segment corresponding to a full OER. In the ProvOER Model, we set the combination between two Segments to facilitate the organization and monitoring of this activity. That's because the number of resources combined may vary.

This way, the provenance documentation is carried out in stages. The Segments are grouped, two by two, until all the combinations performed to create the new OER are registered. In this sense, we maintain, for an OER, an attribute called status, which can assume the values *Intermediate* and *Finalized*. An intermediate OER is a resource that is not finished and available for use, as it is not the final result of the remix but rather one of the blending steps. Still, it is stored for provenance purposes, and the user can view its metadata. An OER whose status is finalized corresponds to the resource created at the end of the remix activity.

In a remix activity, it is essential to consider the particularities related to the author and Segment. The new OER created from the remix activity is authored by one or more authors, which may or not coincide with the authors of the original OER. Thus, the authors of the combined OER and the created OER must be adequately identified for attribution of responsibility and authorship. A situation that should be noted is when an author revises a Segment that is not his/her own and then uses that Segment in a remix activity. In this case, the person responsible for adapting the Segment is credited as the author of that resource. Still, the original Segment can be observed since it is not discarded or overwritten.

The remix of a Segment is subject to the open license assigned to the OER that contains it. For example, an OER licensed through a CC license of type CC BY-ND or CC BY-NC-ND cannot be changed or combined, as this type of license makes it impossible to perform these actions. In addition, for the remix of Segments from OER submitted to different open licenses, it is important to check the compatibility between the licenses and which rules must be followed for the full OER licensing. Thus, knowing and registering the permissible license of the original Segments becomes fundamental to perform the remix activity. We also point out that the revise and remix activities do not change the open license of the original OER.

As previously described, an OER and a Part of OER are formed by at least one Segment corresponding to the full resource. Thus, in terms of OER/Part of OER and the Segments that compose it, we can point to the combination between (i) OER, (ii) Parts of OER, (iii) Segments, (iv) OER and Parts of OER, (v) OER and Segments, (vi) Parts of OER and Segments and (vii) OER, Parts of OER and Segments.

We can also highlight the possibility of creating a new OER through the revise and/or remix of two or more original OER. In this case, all the considerations described above for revise and remix activities must be considered.

## 6 Results and discussion

**Figure 3:**
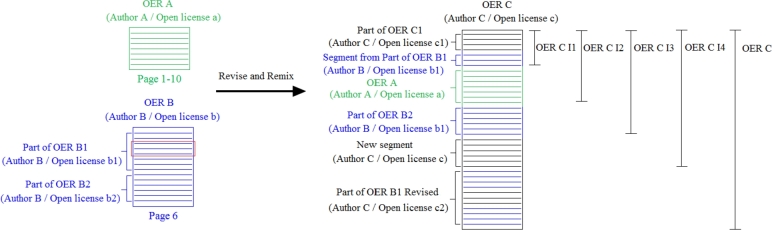
Case study for the validation of the ProvOER Model. The OER C was created by remixing new content, Segments from Part of OER B1, original OER A, Part of OER B2, and Part of OER B1 revised through an insert action. Part of OER B1 and B2 make up the original OER B. The black continuous stretch of OER C correspond to a new content created by author C.

In this section, we present the example performed for validating the ProvOER Model, and a discussion of the results. This case study explains how the ProvOER Model allows the documentation and following of the consecutive transformation processes that result in an OER. For the correct and complete description of the provenance of OER, it is important to store all the metadata in a database. All tables were created in Oracle Live SQL according to the Entity-Relationship model specifications. Fig. 3 shows an example of an OER created through revise and remix activities.

To identify the provenance information of the OER C is necessary to document the steps that were performed to create this resource. In this case, an adaptation and successive combinations of Segments and Segments from OER A and OER B. Original OER A was created by author A, and it is licensed under an open license a. This resource begins on page 1 and ends on page 10. Original OER B was created “from scratch” and it is divided into Parts of OER called B1 and B2. OER B and its components were created by author B but are subject to different open licenses. For validation purposes, the open licenses a and b do not present restrictions for adaptation and combination. OER C has an author named C and is licensed under an open license c.

One aspect that must be documented is the continuous stretch, which delimits the beginning and end of a Segment in an OER. An OER/Part of OER comprises several Segments, so the Segment used in the revise and/or remix activity must be identified and located in the document for provenance purposes. In the example, to facilitate the graphic illustration and explanation, we defined that the granularity of a Segment is a line. For instance, the Part of OER B1 starts at line 1 and ends at line 8 and, therefore, has a length of 8 lines. In the ProvOER Model, for the documentation of the revise and remix activities, all the Segments that make up the OER must be stored in the database.

**Figure 4:**
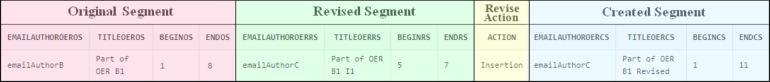
Instantiation of metadata referring to the revise activity, according to the ProvOER Model. The first four fields in the table correspond to the original Segment (Part of OER B1), while the following four fields correspond to the Revised Segment (Part of OER B1 I1). The other field corresponds to the Review Action (insertion), and the last four fields present information about the OER created (Part of OER B1 Revised).

One of the components of the OER C is Part of OER B1 Revised, which is the result of the revision of Part of OER B1 from the OER B. This Part of OER is located on page 6 of OER B. The revision action carried out was the insertion. Fig. 4 shows the table revise created from the ProvOER Model to describe this activity. The table revise is organized as follows: the first four columns correspond to the information about the Original Segment (OS), the other four columns are kept for the Revised Segment (RS), and the last columns allow the identification of the Created Segment (CS) at the end of the revise activity. CS corresponds to a full OER. For each of these Segments is stored: the email of the author of the OER which contains the Segment, the title of the OER, and the continuous stretch that delimits the Segment.

The Original Segment corresponds to Part of OER B1, which starts and ends on lines 1 and 8 of OER B. As illustrated in Fig. 3 by the red rectangle, a Segment of size three, which begins at line 5 and ends at line 7, was inserted in Part of OER B1. To document this action was created an intermediate OER named Part of OER B1 I1. This resource is a copy of Part of OER B1 since the original Segment is not overwritten. At the end of this action, Part of OER B1 Revised was created. It should also be noted that the Segment inserted in Part of OER B1 is new and created by author C, as the OER Part B1 Revised. In the ProvOER Model, the original Segment, the revise action, and the location in which the revise was carried out can be identified, which is essential for the description of the provenance of an OER created by revise activity.

**Figure 5:**
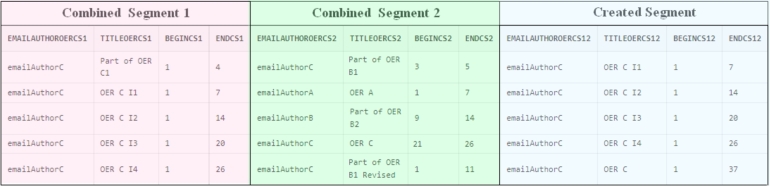
Instantiation of metadata referring to the remix activity, according to the ProvOER Model. The first four fields in the table correspond to the combined Segment 1, the following four fields correspond to the combined Segment 2, and the last four fields present information about the OER created (OER C).

As we pointed out in Section 3, the remix activity is stored in stages, with the Segments coming from different OER being combined successively two by two resources. This way of combination helps the organization because it facilitates the understanding and identification of the remixed Segments. Thus, until the resource is completely created, an OER labeled as “intermediary” is created at the end of each step. For provenance control, we have established that the title of an intermediate OER must be: the title of the created OER + I + number of the combination step. For example, the “OER C I1” results from the first combination step, in which the Part of OER C1 and a Segment from Part of OER B1 were remixed. Fig. 5 presents the table that stores the data referring to a remix activity, according to the ProvOER Model.

The first four columns of the table remix in Fig. 5 correspond to information referring to Combined Segment 1 (CS1). The other four columns correspond to Combined Segment 2 (CS2), and the last columns store the information about the Created Segment (CS12) by combining CS1 and CS2. We must point out that CS12 is a new OER. The size of the OER created at the end of each step of the combination corresponds to the size of CS1 plus the size of CS2. For all Segments, we store the email of the author of the OER that contains the Segment, the title of the OER, and the size of the continuous stretch that delimits the Segment.

OER C was created in five steps. In the first step, Part of OER C1 was combined with a Segment from Part of OER B1, which begins at line 3 and ends at line 5. The OER created was OER C I1. In the second step, OER C I1 was combined with OER A to create OER C I2. Next, this resource was remixed with the Part of OER B2 to create the OER C I3. This intermediate resource was combined with a new Segment created by the author of OER C, which starts at line 21 and ends at line 26 of OER C. Finally, OER C I4 was combined with the Part of OER B1 Revised to create the OER C. We can highlight that all the combined Segments and respective continuous stretches are documented, which makes it possible to identify how the final OER was created. In addition, we can correctly identify the order in which the combinations were performed.

We want to highlight the importance of identifying the author responsible for authoring a Segment. In this sense, for creating an OER, we must consider the possibility of inserting a new Segment or a Segment from another OER. In the first case, we can illustrate the creation of Part of OER B1 Revised, in which a Segment of length three was inserted in Part of OER B1. The inserted Segment was new and authored by author C, as well as Part of OER B1 Revised and the OER C. The other Segments that compose Part of OER B1 were authored by author B. In the second case, we can highlight using a Segment from the Part of OER B1 in the first step of the remix activity. This Segment, unlike OER C, was created by author B. The responsible for the new Segment must be referenced for the contribution. At the same time, the unaltered stretch must be attributed to the original author.

Although the validation focuses on a case study, it is essential to highlight that the ProvOER Model can be associated and applied to any digital environment that allows the creation of new OER from the resources stored in an environment, such as a digital repository. In a digital repository, numerous and different transformation processes can be carried out to create an OER, making it difficult and unfeasible to manually fill in the minimum metadata set that makes up the ProvOER Model. In addition, the specificity of the metadata that make up the ProvOER Model, such as the size of the continuous stretch, may discourage filling them in. In this sense, semi-automatic metadata filling is a more practical and motivating strategy.

By associating the ProvOER Model with a digital repository, metadata, such as those related to the author, OER title, and open license, can be automatically collected based on the metadata stored in the digital environment. If this information is not available, the collection is manual. Metadata describing revision and remixing can be collected from software or API that compares and indicates the difference between two textual resources. In this case, the software or API must be associated with the digital environment. We emphasize that it is essential that the software or API presents metadata that makes it possible to identify the granularity and size of the continuous stretch of the original OER that was subjected to adaptation and/or combination. In the ProvOER Model, this information is necessary to describe a review and remix activity.

A simplified strategy, which does not consider software or API external to the digital environment, is to set the granularity of an adapted and/combined Segment as a line and make comparisons based on this information. It is possible to develop an algorithm in which the line number is automatically identified. In this case, we can perform the following analyses:

• OER created through Revision - Insert Action: Comparison between all lines of the revised OER and the lines that make up the original OER. All lines that are present in the revised OER but not found in the original OER are the result of an insert;
• OER created through Revision - Removal Action: Comparison between all lines of the original OER and the lines of the revised OER. Lines not present in the revised OER were removed;
• OER created through Remix: Comparison between all lines of the remixed OER and the lines of the original OER. The lines found in the remixed OER and in a given original OER correspond to the combined continuous stretch.

As noted in Section 4, the metadata standards Dublin Core, IEEE LOM and LRMI, and digital repositories Connexions and OER Commons allow the description of the provenance of OER. Thus, for comparison purposes, we defined the following parameters related to the provenance of the OER: (i) author, (ii) formation of the resource, (iii) original OER, (iv) revise action, (v) location of the referring revise, and (vi) remix activity.

The first parameter compared is the author, corresponding to the person responsible for creating a new OER. This information is essential, as new resources can be created from OER from different authors. Thus, authorship must be warmly identified and attributed. For example, for an OER created by combining other resources, the author of the full OER and each combined resource must be identified and held responsible for the contribution.

The author can be documented in the Dublin Core by the metadata *creator*. In the IEEE LOM, information about the author composes the *Life Cycle* category, in which the relevant metadata for documenting the data provenance is specified. In the standard, the author identification is recommended. In LRMI, there is no class or property to document the author of the educational resource. An OER can be created individually or collaboratively in the Connexions digital repository. In this sense, all those responsible for this action are identified. A weakness that should be pointed out for OER Commons is that information about the author is not collected but automatically assigned when the user, who is connected to the repository, fills in the metadata required for the resource description. The person responsible for filling in the metadata is considered the author. However, this situation is not always valid. In the ProvOER Model, the person responsible for creating an OER is identified through an email, name, and URL.

The second parameter corresponds to the creation of an OER from other resources that have different open licenses. This particularity must be recorded. Through the Dublin Core and IEEE LOM standards, it is possible to identify the *rights* regarding the use conditions of the educational resource. However, there is no metadata to document the composition of an OER. From our point of view, as these metadata standards are not specific to OER documentation, this particularity is not dealt. This limitation is also present in LRMI and the digital repositories. OER is commonly understood as a single block of knowledge, but it is essential to believe that one or more independent OER can make up a resource. In this sense, we define in the ProvOER Model the elements Segment and Part of OER, which allow the documentation of this scenario.

The third aspect compared is the possibility of identifying the original OER, used as a basis for creating a new resource. As explained in Section 4, the metadata standards and digital repositories allow the documentation of this information. In Connexions and OER Commons, the original OER can be accessed and viewed through a link available in the current OER. However, in these repositories, only one OER can be referenced as the original OER, which is a barrier to describing a remix activity that combines two or more resources. In the ProvOER Model, it is possible to store and document all original OER that contributed to creating a new resource.

The other points of comparison are related to the revise activity. The first comparison refers to identifying the type of revision action performed, such as an insertion or a translation. In Dublin Core, IEEE LOM, and LRMI, there is no metadata to document the process of transforming. In the Connexions, a revise action is recorded when a version of a feature or derivative work is created. In OER Commons, the revise action cannot be specified. In the ProvOER Model, the revise action is stored through metadata called “action”. Although the actions in this article are restricted to text, others can also be considered, as long as the metadata necessary for the documentation of a particular action is added in the ProvOER Model. For example, a revise action that can be pointed out is changing the resolution of images and videos. Thus, it is essential to maintain for a Segment an attribute called “resolution”.

We also compared the possibility of locating the continuous stretch in the original OER where the revision was carried out. We consider the localization of the page on which the revise activity was performed, which makes it easier to find the continuous stretch reviewed. This information may also refer to the integral OER when the resource is completely revised, such as a translation. In addition, for provenance purposes, it is essential to correctly identify the beginning and the end of the adapted continuous stretch. This aspect is also important for the attribution of authorship. In the ProvOER Model, we establish the continuous stretch attribute that delimits the size of a Segment and allows its stretch to be related to different granularities. In metadata standards and digital repositories, it is impossible to identify and document the location of a revision.

Finally, we compare the possibility of documenting a remix activity. Therefore, it is essential to identify the combined OER stretch and the order in which this activity was carried out. Although Dublin Core, IEEE LOM, Connexions, and OER Commons provide original OER documentation, it is impossible to specify the stretch used in a combination. Also, as pointed out earlier, it is impossible to document the remix of two or more resources in the digital repositories. On the other hand, in the ProvOER Model we established a specific attribute for registering the OER stretch used in a remix activity. Furthermore, as we recorded this activity in steps combining successively two resources, it is possible to represent the order in which the combinations were performed. We summarize all comparisons discussed in this section in Table 1.

**Table 1:** Comparison among the metadata standards Dublin Core, IEEE LOM and LRMI, digital repository Connexions and OER Commons, and the ProvOER Model, as for the parameters author, formation of the resource, original OER, revise action, location of the referring revise, and remix activity.

	Dublin Core	IEEE LOM	LRMI	Connexions	OER Commons	ProvOER Model
Author	X	X		X	X	X
Formation						X
Original OER	X	X	X	X	X	X
Revise (action)				X		X
Revise (location)						X
Remix						X

As can be seen from Table 1, some of the provenance metadata that make up the ProvOER Model are common to the metadata standards Dublin Core, IEEE LOM and LRMI and digital repository Connexions and OER Commons, such as, original OER and author information. In addition to this information, we consider other metadata that are present in metadata standards and digital repositories but are not considered for provenance purposes. For example, due to the possibility of revision, language impacts the documentation of the history of an OER. In addition to these metadata, we can point out the attributes open license and public domain as peculiarities inherent to the context of OER. It is important to point out that the detailing of the Segment used as a basis for creating a new OER and the revision and/or remix metadata are exclusive contributions of the ProvOER Model. Through this new metadata layer it is possible to specify the granularity and size of the revised and/or remixed continuous stretch and describing the activities that resulted in a new resource.

## 7 Conclusion and future work

Open Educational Resources allow the creation and sharing of educational knowledge. Due to the characteristic of legal openness, new OER can be created from the revision (adaptation) of an OER and/or the remix (combination) of different OER. Due to the possibility of creating a resource from others, the user must have enough information about the transformation processes carried out so that OER can be used safely and reliably. Thus, the data provenance has a fundamental role in describing the history of an OER, from its origin to its current state.

This article proposes the ProvOER Model, a Provenance Model for Open Educational Resources, which allows the documentation and identification of the provenance of an OER. The ProvOER Model was designed according to the Entity-Relationship Diagram, which allows the representation of all the relevant components for the description of the history of an OER. In addition, we have established a set of metadata that reflects the particularities of OER and the activities that was used to build a new resource.

In the literature, no related work focus their efforts on documenting the provenance of OER. We identified examples of metadata standards and digital repositories that make it possible to partially describe the provenance of an OER. However, some limitations do not allow the correct and complete description of the provenance of OER.

Through the ProvOER Model, it is possible to document the actions that were used to compose a new OER and track the order in which these activities took place. We also highlight the possibility of locating the continuous stretch of an OER that was adapted or combined to create a resource. This information is critical for documenting a revise and a remix activity and attributing authorship to the original OER and the current OER.

As future work, we highlight the importance of associating the ProvOER Model with a digital environment and enabling the semi-automatic collection of the minimum set of metadata from the OER. We also consider that the ProvOER Model can be used as a basis for elaborating an approach for assessing the quality of OER based on data provenance. Due to the possibility of creating new resources from original OER of different quality levels and coming from various sources, it is essential to provide means to identify the OER created. Thus, identifying data provenance becomes crucial for evaluating the quality of an OER.

## Declaration of Competing Interest

The authors declare no competing interests.

## Footnotes

1 https://livesql.oracle.com/.

2 https://creativecommons.org/.

3 https://www.gnu.org.

4 https://opendatacommons.org/.

5 https://www.dublincore.org/specifications/lrmi/.

6 https://cnx.org/.

7 https://www.oercommons.org/.
